# The Autofluorescence Patterns of *Acanthamoeba castellanii, Pseudomonas aeruginosa* and *Staphylococcus aureus*: Effects of Antibiotics and Tetracaine

**DOI:** 10.3390/pathogens10070894

**Published:** 2021-07-14

**Authors:** Hari Kumar Peguda, Saabah B. Mahbub, Tashi Doma Sherpa, Dinesh Subedi, Abbas Habibalahi, Ayad G. Anwer, Zi Gu, Mark D. P. Willcox, Ewa M. Goldys, Nicole A. Carnt

**Affiliations:** 1 School of Optometry and Vision Science, University of New South Wales, Sydney 2052, Australia; tashy422@gmail.com (T.D.S.); dinesh.subedi@monash.edu (D.S.); m.willcox@unsw.edu.au (M.D.P.W.); n.carnt@unsw.edu.au (N.A.C.); 2 ARC Centre of Excellence for Nanoscale Biophotonics, Graduate School of Biomedical Engineering, University of New South Wales, Sydney 2052, Australia; s.mahbub@unsw.edu.au (S.B.M.); a.habibalahi@unsw.edu.au (A.H.); a.anwer@unsw.edu.au (A.G.A.); e.goldys@unsw.edu.au (E.M.G.); 3 School of Biological Sciences, Monash University, Clayton 3800, Australia; 4 School of Chemical Engineering, University of New South Wales, Sydney 2052, Australia; zi.gu1@unsw.edu.au

**Keywords:** *Acanthamoeba*, cornea, bacteria, autofluorescence, keratitis

## Abstract

*Acanthamoeba* Keratitis (AK) can lead to substantial vision loss and morbidity among contact lens wearers. Misdiagnosis or delayed diagnosis is a major factor contributing to poor outcomes of AK. This study aimed to assess the effect of two antibiotics and one anaesthetic drug used in the diagnosis and nonspecific management of keratitis on the autofluorescence patterns of *Acanthamoeba* and two common bacteria that may also cause keratitis. *Acanthamoeba castellanii* ATCC 30868, *Pseudomonas aeruginosa* ATCC 9027, and *Staphylococcus aureus* ATCC 6538 were grown then diluted in either PBS (bacteria) or ¼ strength Ringer’s solution (*Acanthamoeba*) to give final concentrations of 0.1 OD at 660 nm or 10^4^ cells/mL. Cells were then treated with ciprofloxacin, tetracycline, tetracaine, or no treatment (naïve). Excitation–emission matrices (EEMs) were collected for each sample with excitation at 270–500 nm with increments in 5 nm steps and emission at 280–700 nm at 2 nm steps using a Fluoromax-4 spectrometer. The data were analysed using MATLAB software to produce smoothed color-coded images of the samples tested. *Acanthamoeba* exhibited a distinctive fluorescence pattern compared to bacteria. The addition of antibiotics and anaesthetic had variable effects on autofluorescence. Tetracaine altered the fluorescence of all three microorganisms, whereas tetracycline did not show any effect on the fluorescence. Ciprofloxacin produced changes to the fluorescence pattern for the bacteria, but not *Acanthamoeba*. Fluorescence spectroscopy was able to differentiate *Acanthamoeba* from *P. aeruginosa and S. aureus* in vitro. There is a need for further assessment of the fluorescence pattern for different strains of *Acanthamoeba* and bacteria. Additionally, analysis of the effects of anti-amoebic drugs on the fluorescence pattern of *Acanthamoeba* and bacteria would be prudent before in vivo testing of the fluorescence diagnostic approach in the animal models.

## 1. Introduction

*Acanthamoeba* keratitis (AK) is a severe sight-threatening condition, predominantly occurring in contact lens wearers [1,2]. Visual outcomes from AK depend on early diagnosis [3]. Furthermore, AK is often misdiagnosed (approximately 50% of the cases), with exacerbation of the infection and vision loss as a result [4]. Culturing of *Acanthamoeba* in the laboratory is considered the gold standard for diagnosis [5]. However, microbial culture is only positive in approximately 55% of cases [6] and a small amount of sample available form smears may not be sufficient to see the protozoa. The ability to differentiate *Acanthamoeba* from more common causes of microbial keratitis could be useful in the diagnosis of AK. This is particularly critical given that bacteria are a much more frequent cause of keratitis than *Acanthamoeba* [7,8] and the treatment differs.

Due to the poor success rate with *Acanthamoeba* culture, alternative methods of diagnosis have been examined including the use of the polymerase chain reaction (PCR) to specifically amplify the DNA of *Acanthamoeba* and, in vivo confocal microscopy (IVCM) to visualize the amoeba in the cornea [6,9,10,11,12]. Culture, PCR, and IVCM can be time-consuming, costly, or difficult to implement in resource-poor settings.

Autofluorescence imaging has been used in many areas of medicine [13,14]. In ophthalmology, autofluorescence imaging is widely used to diagnose and monitor disease, especially diseases of the retina [15,16]. *Acanthamoeba* cells [17] and bacteria [18] produce detectable autofluorescence and an earlier study [17] has shown that *Acanthamoeba* exhibit a unique autofluorescent signature compared to bacteria. Previous studies hypothesised that amino acids such as tryptophan, tyrosine, phenylalanine, and coenzyme nicotinamide adenine nucleotide hydride (NADH) were responsible for the difference in autofluorescence and its intensity [17,19], with tryptophan being particularly important [17]. This offers scope to explore novel differential diagnostics for *Acanthamoeba* keratitis. The primary objective of this study was to assess the autofluorescence pattern of *Acanthamoeba castellanii* and to examine its differentiating features from two bacterial types that are more commonly isolated from keratitis.

We also evaluated the effect of two local antibiotics and one local anaesthetic drug (tetracycline, ciprofloxacin, and tetracaine) treatment on the autofluorescence pattern of these three microbes. These agents are commonly used in the early nonspecific management and diagnostic workup prior to a definitive diagnosis. We hypothesised that these agents would exhibit different effects due to their mode of action.

## 2. Results

**Figure 1:**
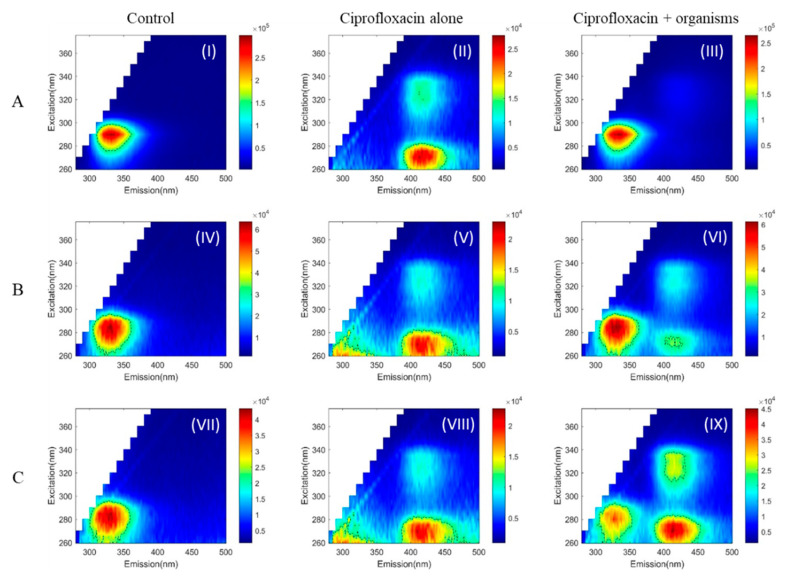
Autofluorescence pattern of *Acanthamoeba Castellanii* (**A**, **I**–**III**), *Pseudomonas aeruginosa* (**B**, **IV**–**VI**), and *Staphylococcus aureus* (**C**, **VII**–**IX**). Control—organisms alone (**I**,**IV**,**VII**), ciprofloxacin alone (**II**,**V**,**VIII**), and organisms with ciprofloxacin (**III**,**VI**,**IX**). Full width half maximum (black dashed line) shows the shape of EEM/each peak calculated. Ciprofloxacin test concentration—0.00625 μg/mL.

**Figure 2:**
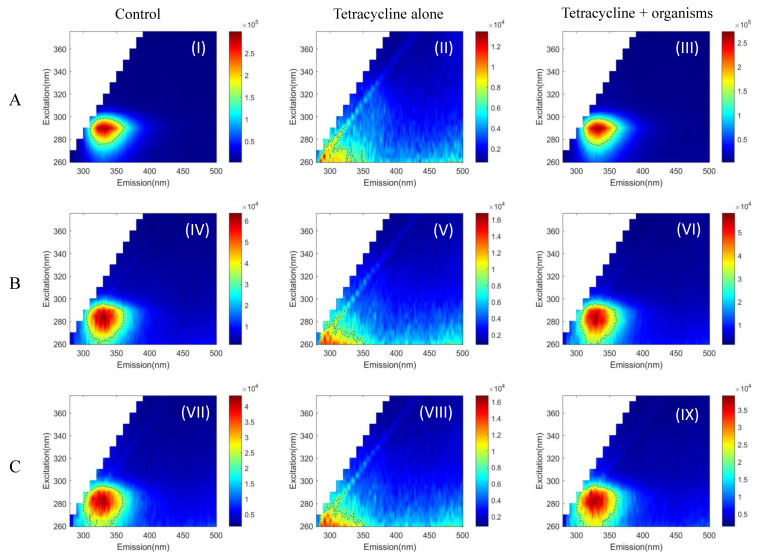
Autofluorescence pattern of *Acanthamoeba Castellanii* (**A**, **I**–**III**), *Pseudomonas aeruginosa* (**B**, **IV**–**VI**), and *Staphylococcus aureus* (**C**, **VII**–**IX**). Control—organisms alone (**I**,**IV**,**VII**), tetracycline alone (**II**,**V**,**VIII**), and organisms with tetracycline (**III**,**VI**,**IX**). Full width half maximum (black dashed line) shows the shape of EEM/each peak calculated. Tetracycline test concentration—0.0125 μg/mL.

**Figure 3:**
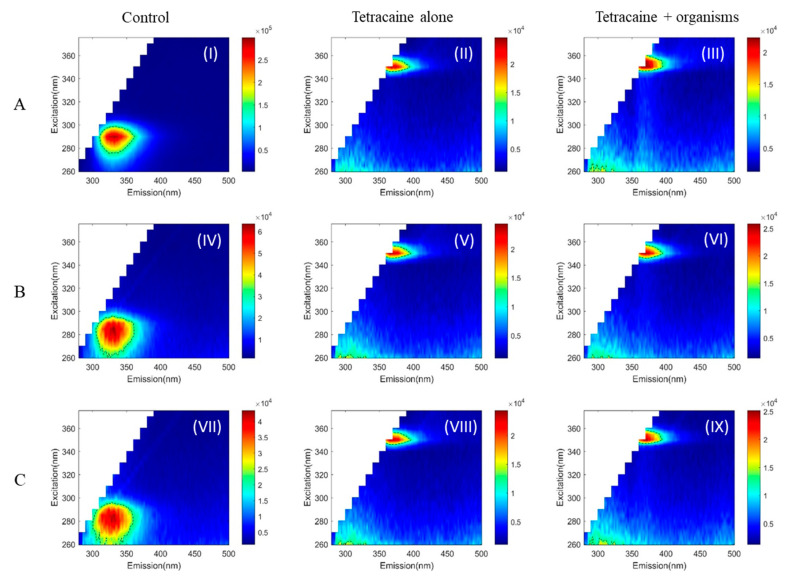
Autofluorescence pattern of *Acanthamoeba Castellanii* (**A**, **I**–**III**), *Pseudomonas aeruginosa* (**B**, **IV**–**VI**), and *Staphylococcus aureus* (**C**, **VII**–**IX**). Control—organisms alone (**I**,**IV**,**VII**), tetracaine alone (**II**,**V**,**VIII**), and organisms with tetracaine (**III**,**VI**,**IX**). Full width half maximum (black dashed line) shows the shape of EEM/each peak calculated. Tetracaine test concentration—10 µg/mL.

The two-dimensional autofluorescence pattern in the EEMs measured for the three microorganisms without drugs is shown in Figure 1, Figure 2 and Figure 3: I, IV, VII. Truncated EEM images are presented (excitation 260 to 375 nm and emission 280 to 500 nm), as no autofluorescence features there found outside of this region. To quantify the observations, the peak position and full width half maximum (FWHM) for each observed peak in the EEM were calculated to determine details of its spectral location and shape and allow comparisons of the shape. The FWHM is drawn (black dashed line) as a contour over the EEM (Figure 1, Figure 2 and Figure 3).

*Acanthamoeba* exhibited a single autofluorescence peak at (excitation/emission: 290 nm/336 nm). The EEM (Figure 1, Figure 2 and Figure 3 (I)) shows that *Acanthamoeba* had a more oval-shaped autofluorescence peak; (at FWHM, the excitation and emission ranged from 276 to 299 nm and 310to 362 nm, respectively) compared to both bacteria (Figure 1, Figure 2 and Figure 3 (IV,VII)). The autofluorescence peaks of *Pseudomonas aeruginosa* (Figure 1, Figure 2 and Figure 3 (IV)) and *Staphylococcus aureus* (Figure 1, Figure 2 and Figure 3 (VII)) were approximately round with slightly different peaks (*P. aeruginosa*: 285 nm/332 nm and *S. aureus*: 280 nm/328 nm). In bacteria, the FWHM of EEM spreads across ~60 nm for both excitation and emission.

The EEM of drugs was examined initially with the appropriate solvent. Ciprofloxacin with Ringer’s solution gave two peaks, where the dominant peak was located at (270 nm/412 nm) (Figure 1 (II)). Ciprofloxacin with PBS has similar autofluorescence features with a dominant peak at (270 nm/410 nm) (Figure 1 (V,VIII)). Tetracaine had a very strong peak at (350 nm/368 nm) in both solvents (Figure 3 (II,V,VIII)), but tetracycline did not have any characteristic peaks (Figure 2 (II,V,VIII)). The EEM range was limited to avoid second-order Rayleigh scattering, especially for the tetracaine peak.

*Acanthamoeba* had a 10-fold stronger signal compared to ciprofloxacin alone, and that is why peaks from ciprofloxacin were barely visible (Figure 1 (III)). *P. aeruginosa* and *S. aureus* gave similar levels of signals with ciprofloxacin. *P. aeruginosa* lowered the dominant ciprofloxacin peak (270 nm/412 nm) (Figure 1 (VI)), but both peaks remained stable for *S. aureus* (Figure 1 (IX)).

With the use of tetracycline, all samples preserved their original peaks and peak widths (Figure 2 (III,VI,IX)). However, the characteristic fluorescence peaks were absent with the use of tetracaine in all samples (Figure 3 (III,VI,IX)).

## 3. Discussion

This study has confirmed that *Acanthamoeba* can be differentiated from bacteria (i.e., *P. aeruginosa* and *S. aureus*) based on its autofluorescence pattern [17] in vitro, and has shown that, for bacteria only, the autofluorescence pattern changed after exposure to ciprofloxacin. Previously, EEM patterns have been used to differentiate *Acanthamoeba* from other bacteria, *Escherichia coli*, *Elizabethkingia miricola*, *Achromobacter ruhlandii*, as well as *S. aureus*, *P. aeruginosa*, and the yeast *Candida albicans* [17]. The authors reported that *Acanthamoeba* appeared distinct from other organisms by having a ‘comma-shaped EEM’. However, the EEM was more oval shaped in the current experiments. This difference may be due to the different *Acanthamoeba* strains, i.e., *A. castellanii* ATCC50370 [17] compared to *A*. *castellanii* ATCC30868, in the current study and the instruments used in the studies, even though similar parameters were used [17]. However, the results presented in the current study may be more reliable as the spectra collected were corrected (using FluorEssence^TM^ software) for the lamp intensity and excitation monochromator, while the spectra in the previously published study were not, hence the earlier data could have been affected by instrumental artifacts. Additional *Acanthamoeba* strains (both ATCC and environment samples) need to be studied to determine whether a similar comma or oval-shaped signature pattern exists across all the strains.

The bacterial EEM in the previous publication [17] was similar to those in the current paper, i.e., circular, although the EEM of *P. aeruginosa* had a second maximum at approximately 440 nm/435 nm in the previous publication [17]. Again, this difference may be attributed to the use of different strains, instruments, or correction factors. Further research is warranted to resolve this issue.

Common ocular drugs can be of potential use in distinguishing *Acanthamoeba* from other bacteria based on the EEM pattern. The rationale for using these drugs was due to their different mode of action and frequent use in the diagnostic workup and early nonspecific management of keratitis. Ciprofloxacin acts on bacterial DNA gyrase and polymerase and inhibits cell division, whereas tetracycline inhibits prokaryotic protein synthesis [20]. Tetracaine has inhibitory, antibacterial, and anti-amoebic action, with the latter action being through disruption of the protozoan cell membrane leading to cell lysis [21].

Previous studies on fluorescence emission of both ciprofloxacin and tetracaine have shown similar results to those in the current study with maximum fluorescence observed at 440 and 357 nm, respectively [22,23]. Bacteria that are sensitive to ciprofloxacin exhibit a 10-fold increase in autofluorescence at the single wavelength of 420 nm compared to resistant bacteria [24]. Further research is required to determine whether the EEM of antibiotic sensitive and resistant bacteria are different.

Several studies have shown that topical anaesthetics have inhibitory and antibacterial effects on ocular microbes [25,26]. Tetracaine is active against *Acanthamoeba, S. aureus,* and *P. aeruginosa* [27,28,29]. This current study demonstrated the effect of tetracaine on the EEM of both bacteria and amoeba by eliminating the fluorescence signal.

The current results confirmed that EEM can differentiate *Acanthamoeba* from two common ocular bacteria and have shown that ciprofloxacin can alter the EEM of the bacteria. Additionally, this alteration in EEM pattern with ciprofloxacin can differentiate *Acanthamoeba* with one characteristic bright visual peak compared to the two bacteria which exhibited three visible peaks. Polymicrobial keratitis of *Acanthamoeba* and bacteria is diagnosed occasionally, 5/111 patients in one study [30], and it would be interesting to examine the EEM combinations of bacteria with *Acanthamoeba*. What may happen is that the peaks partially superimpose, giving unique signatures depending on the combinations of microbes examined, and thus should be examined in future studies.

We can use a more objective way of distinguishing the organisms by measuring the magnitude difference in the fluorescence signal. In our case, it is not possible due to the differences in the fluorescence peaks of tetracycline or tetracaine in comparison to the organisms treated. The peak of tetracycline is 20-fold weaker than *Acanthamoeba Castellanii* (Figure 2A (I–III)), and the peak of tetracycline is ~5-fold weaker than *Pseudomonas aeruginosa* (Figure 2B (IV–VI)), and *Staphylococcus aureus* (Figure 2C (VII–IX)). That is the reason we could not see any peak of tetracycline within tetracycline + organisms plots (Figure 2 (III,VI,IX)). However, the peak of tetracaine is 1/3 of the sample, and we should see the organism’s peak. However, we think tetracaine is toxic to the organisms tested and kills the fluoresce signal of the organisms even though the concentration used is less than reported [28]. Tetracaine in particular is more toxic compared to proxymetacaine, oxybuprocaine on the trophozoites, and cysts of *Acanthamoeba* spp. This study investigated the autofluorescence pattern of protozoa and two bacteria and these results need to be further explored by in vitro experiments on different strains of *Acanthamoeba* and bacteria before translating into animal model. Since *Acanthamoeba* exists in two life forms, trophozoites and cysts, assessment of the autofluorescence pattern of both forms is also needed, especially as both forms can be found during microbial keratitis [31]. The topical drugs used for the treatment of AK such as polyhexamethylene biguanide (PHMB), chlorhexidine, brolene, hexamidine, and miltefosine could also be investigated. These frontline drugs for AK were not considered in the current experiments as the goal of the current research was to explore possible effects of commonly used or prescribed topical drugs by GP or local ophthalmologist on autofluorescence patterns before visiting tertiary care or an ophthalmologist specialised in corneal diseases. These current results can be further explored by measuring the EEM on corneal scrapes/biopsies along with studying factors, such as number of organisms, amount of sample and influence of background fluorescence, that are required to achieve a positive signal. As excitation wavelengths below 400 nm can be harmful to ocular structures, it would be prudent to use non-invasive, ultraviolet-free techniques such as autofluorescence multispectral imaging [32,33] in future experiments.

## 4. Materials and Methods

### 4.1. Culturing of Acanthamoeba and Bacteria

*Acanthamoeba castellanii* ATCC 30868 was cultured in peptone yeast glucose (PYG) medium (ATCC medium: peptone—1.25 g/L, yeast extract—1.25 g, dextrose—3.0 g/L with pH 6.5) at 32 °C for 3–5 days until trophozoites reached 90% confluency. Subsequently, trophozoites were washed in freshly prepared in ¼ strength Ringer’s solution 3 times by centrifuging the sample at 500× *g* for 10 min. This step was implemented to avoid any fluorescence from the growth media as has been described previously [17]. The final concentration of the *Acanthamoeba* cells was adjusted to 10^4^ cells/mL using a Neubauer hemocytometer (Hirschmann, Eberstadt, Germany). *Pseudomonas aeruginosa* ATCC 9027 and *Staphylococcus aureus* ATCC 6538 were cultured in Tryptic Soy Broth [TSB] (Becton, Dickinson and Company, New Jersey, USA) by incubating at 37 °C for 18–24 h. The bacterial cells were collected by centrifugation at 3200× *g* for 10 min and washed three times with phosphate-buffered solution [PBS] (NaCl 8 g/L, KCL 0.2 g/L, Na_2_HPO4 1.4 g/L, KH_2_PO_4_ 0.24 g/L, pH 7.2). The final concentration of the bacteria was adjusted to 10^8^ CFU/mL (0.1 optical density at 660 nm) using an Omega spectrofluorometer (BMG Labtech, Victoria, Australia).

### 4.2. Measurement of Autofluorescence Signals

**Figure 4:**
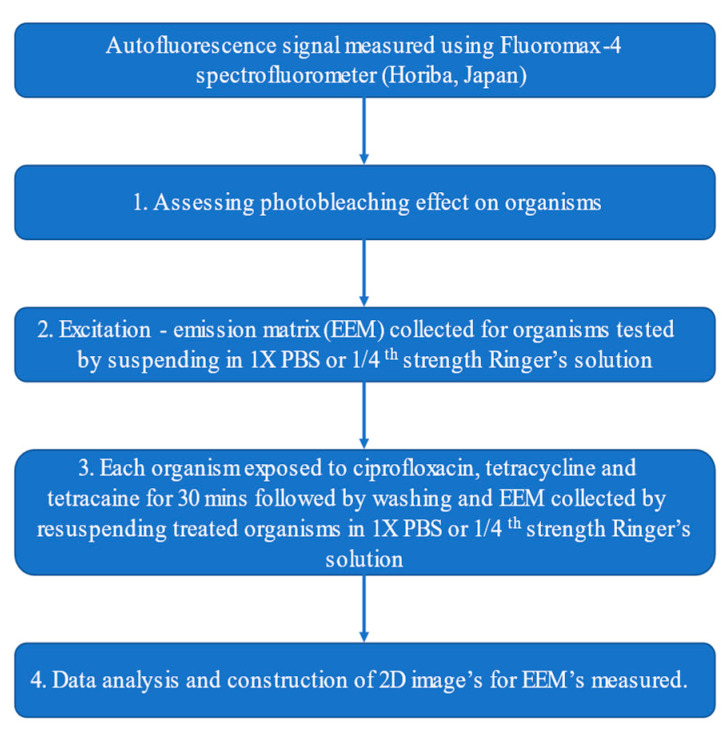
Flow chart shows experimental design.

Autofluorescence signals were measured using an excitation–emission matrix in a Fluoromax-4 spectrofluorometer (Horiba, Tokyo, Japan) and the flow of the experiments is given in Figure 4. Firstly, any photobleaching caused by light exposure of the cells was assessed by measuring emission spectra of *Acanthamoeba castellanii, Pseudomonas aeruginosa,* and *Staphylococcus aureus* from 300 to 550 nm in 2 nm steps whilst exciting each organism at an excitation wavelength of 285 nm. The samples were exposed for 0.5 s in each measurement.

For test measurements, EEMs were collected for each sample (2.5 mL) in triplicate by scanning across 270–500 nm excitation wavelengths with increments in 5 nm steps. Emission signals were examined at 280–700 nm at 2 nm emission wavelength steps. During the EEM collection for the *Acanthamoeba castellanii,* samples were shaken every five-minutes to prevent the sedimentation of the cells. Shaking was not required for *P. aeruginosa and S. aureus* as there was no evidence of sedimentation of these bacterial cells during the experiment. 

### 4.3. Measurement of Autofluorescence Signal after In Vitro Treatment with Topical Medications

We examined the cultures of *Acanthamoeba castellanii* ATCC 30868, *Pseudomonas aeruginosa* ATCC 9027, and *Staphylococcus aureus* ATCC 6538 under the influence of various drugs, used in the diagnosis or early nonspecific management of the disease, as well as microbes without drugs (naïve). Two antibiotics (ciprofloxacin and tetracycline, Sigma-Aldrich, Sydney, Australia), each with a different mode of action, and a local anaesthetic (tetracaine hydrochloride 1% *w*/*v*, Bausch and Lomb, Sydney, Australia) were used. Ciprofloxacin and tetracycline were prepared by dissolving in distilled water using *w/v*% and tetracaine by *v*/*v*% to achieve final concentration. Drugs were applied to cultures one at a time (not simultaneously). To avoid any bactericidal action, minimum inhibitory concentrations (MICs) of antibiotics, as recorded by the clinical and laboratory standards institute (CLSI) for these bacterial species, were diluted by 4-fold, and tetracaine was also diluted to reduce the toxicity on trophozoites [28]. Accordingly, cells were exposed to 0.00625 μg/mL ciprofloxacin, 0.0125 μg/mL tetracycline or 10 µg/mL tetracaine for 30 min at room temperature before measurement of the EEM. An EEM was also collected for each drug alone at its test dilution (2.5 mL) and was used to determine the fluorescence characteristics of the drug. The data were analysed using MATLAB software (Version: 2019b, The MathWorks Inc., Natick, MA, USA) to produce the 2D images of the EEM.

## 5. Conclusions

These preliminary results showed a characteristic autofluorescence pattern with *A. castellanii* which can be used to distinguish it from *P. aeruginosa* and *S. aureus.* The autofluorescence of *Acanthamoeba* and bacteria can be manipulated to differing extents with the drugs. Further investigation of this technology for different strains of *Acanthamoeba* and bacteria as well as effects of anti-amoebic drugs on the fluorescence pattern of *Acanthamoeba* and bacteria would be prudent before in vivo testing of the fluorescence diagnostic approach in the animal models.
